# Positive effects of platelet-rich plasma (PRP) and a *Sanguisorba officinalis* polysaccharide on the proliferation and differentiation of anterior cruciate ligament (ACL) fibroblasts *in vitro*

**DOI:** 10.1080/13880209.2020.1743325

**Published:** 2020-04-07

**Authors:** Hong Zheng, Wenya Huang, Bing He, Hongchang Tan, Pingzhi Lin, Zhengang Zha

**Affiliations:** a Institute of Orthopedic Diseases and Department of Bone and Joint Surgery, The First Affiliated Hospital of Jinan University, Guangzhou, China; b Department of Orthopedic Surgery, Affiliated Hospital of Guangdong Medical University, Zhanjiang, China; c Department of Orthopedic Surgery, The People’s Hospital of Leizhou, Leizhou, China; d Department of Nursing, Affiliated Hospital of Guangdong Medical University, Zhanjiang, China

**Keywords:** Degenerative joint disease, osteoblastic proliferation, migration and differentiation, apoptosis, TLR-4/NF-κB signalling pathway

## Abstract

**Context:**

*Sanguisorba officinalis* L. (Rosaceae), a famous traditional Chinese medicine. It was recently reported that its polysaccharide could facilitate collagen production.

**Objectives:**

We investigated the mechanism by which *S. officinalis* polysaccharide (SOWPa) and/or platelet-rich plasma (PRP) promote regenerative potential of anterior cruciate ligament (ACL) *in vitro*.

**Materials and methods:**

ACL fibroblasts were treated with SOWPa (25 and 100 mg/kg), PRP, PRP + SOWPa (25 and 100 mg/kg) or vehicle alone for 24, 48, or 72 h. Cell viability, migration ability and apoptosis were evaluated by MTT, transwell and flow cytometry, respectively. Western blot analysis was performed to assess associated protein expression.

**Results:**

PRP, SOWPa (100 mg/kg) or PRP + SOWPa (100 mg/kg) treatment for 72 h significantly improved the cell viability of ACL fibroblasts from 100 ± 7.5% (control) to 156.85 ± 12.82%, 188.08 ± 15.92%, and 223.67 ± 18.82%, respectively, which was evidenced by individual decreased apoptosis rate from 31.26 ± 2.35% (control) to 20.80 ± 1.89%, 18.01 ± 1.55% and 9.33 ± 0.78%. Furthermore, the motility of ACL fibroblasts was significantly improved with increased migrated cell number per field from 5 for control to 26 for PRP, 36 for SOWPa and 44 for PRP + SOWPa, respectively. Moreover, the protein expression of differentiation markers (RUNX2, ALP, BMP2 and Col I) and TLR-4 and phosphorylated p65 (p-p65) was inhibited by the above treatment.

**Discussion and conclusions:**

Data suggested that the addition of SOWPa to PRP increased the regenerative ability of ACL fibroblasts by blocking the TLR-4/NF-κB pathway.

## Introduction

The anterior cruciate ligament (ACL) is an important intraarticular structure to maintain the stability of knee joint and its rupture not only causes pain and knee instability, but also runs the risk of premature degenerative joint disease (Lidén et al. 2008). It is a leading cause of disability in the world, with over 200,000 patients diagnosed with ACL disruptions annually (Kim et al. 2011). Unlike extraarticular ligaments injury, which can be healed even without surgical management (Indelicato 1983), the ACL has an intrinsic inability to heal once injured due to poor vascularization, hence ACL reconstruction with allografts or autografts (hamstring or patella tendon) is the current gold standard of treatment regimen (Petrigliano et al. 2006; Tischer et al. 2007), which definitely poses a major public health and financial burden on affected individuals (Andersson et al. 1991). Though the promising results such as subjective satisfaction and partial stability restoration can be achieved with ACL surgical management, no reliable and satisfactory outcomes in the long term is acquired in follow-up studies (Sun et al. 2016). The increased serious complications including loss of normal knee kinematics and high rates of premature osteoarthritis are observed in clinical treatments (Miller and Gladstone 2002; Strickland et al. 2003; Dopirak et al. 2004). Furthermore, due to slow or incomplete healing, failure of autografts is common with failure rates of 7.2% for patellar tendon grafts and 15.8% for hamstring grafts (Reinhardt et al. 2010). Moreover, owing to the shortage of soft-tissue autografts/allografts tissue, various synthetic, nondegradable allografts or autografts were used in the 1970s and 1980s but were hampered by their inherent limitations such as premature graft rupture, foreign body reactions, osteolysis and synovitis (De Groot et al. 1996). Thus, to overcome these aforementioned problems, there is an increasing interest in strategies to improve the healing of ACL with minimal surgical interference, such as the use of platelet-rich plasma (PRP).

PRP is a high concentrate of autologous platelet and an enriched source of multiple growth factors, such as platelet-derived growth factor (PDGF), platelet-derived epidermal growth factor (PDEGF), platelet-derived angiogenesis factor, transforming growth factor beta (TGF-β), epidermal growth factor, insulin-like growth factor, growth and differentiation factor, and vascular endothelial growth factor (VEGF) (Marx et al. 1998). These cytokines released by PRP can clinically encourage healing of both soft and hard tissues by serving as mitogens, chemoattractants and stimulators of cell proliferation after ACL reconstruction surgeries (Werner and Grose 2003; Kon et al. 2009). Furthermore, PRP activation by collagen amplifies the sustained delivery of these cytokines to injured tissues. Unlike individual recombinant growth factors, PRP can modulate and ‘up-regulate’ one growth factor’s function in the presence of additional growth factors (Smith et al. 2006). However, currently, existing preclinical and clinical studies regarding PRP use for ACL surgery have yielded promising but also controversial results (Hutchinson et al. 2014). In some conditions, single use of PRP failed to demonstrate a clear benefit (Vavken et al. 2011). It is likely due to the degradation of fibrin, one principal extracellular matrix molecule in PRP, in the presence of intra-articular plasmin in the posttraumatic joint (Harrold 1961). Intriguingly, further work in animal models has suggested that collagen–PRP composites exhibited a high efficiency to promote ACL healing (Joshi et al. 2009; Vavken et al. 2012). This may be attributed to the protective effect of the co-polymer against destruction of plasmin on the fibrin in the PRP (Kroon et al. 2002). Although PRP is known to stimulate collagen expression in ACL fibroblasts, its production cannot be guaranteed to impede the degradation of fibrin induced by plasmin. Thus, the use of an adjuvant to promote collagen expression by PRP in the joint may be more effective than supplementation with PRP alone.

Naturally derived polysaccharides are becoming increasingly important and easily accessible source of additives or alternative medicines in food, cosmetics and pharmaceuticals industry due to their biocompatibility, and intrinsic biological properties as compared with synthetic drugs (Sun 2011). Nowadays, emerging evidence suggested that that some polysaccharides have been effectively used to fill space and support the body in regenerating tissue for the treatment of skin and epithelium wounds via enhancement of collagen expression (Xu et al. 2018; Zhang et al. 2018; Slima et al. 2019). One such polysaccharide isolated from the roots of *Sanguisorba officinalis* L. (Rosaceae), has been topically applied to heal wounds, burns and allergic skin diseases (Strickland et al. 2003). Zhang, Chen, et al. (2018) tested the healing efficacy of a purified polysaccharide (SOP) from the roots of *S. officinalis* on burn wound models in mice and histopathological examination of the wound tissues in the SOP-treated animals showed collagen deposition and epidermal formation. The use of this polysaccharide with PRP facilitating collagen expression in the treatment of ALC reconstruction surgeries is therefore strongly suggested. However, there is no available report regarding whether this polysaccharide could facilitate healing of ACL with PRP. Therefore, this study evaluates the collaborative effect of *S. officinalis* polysaccharide (SOWPa), PRP or their combination on the regenerative potential in ACL fibroblasts and to explore the underlying molecular mechanisms.

## Materials and methods

### Materials and chemicals

The dried roots of *S. officinalis* were purchased from Chinese Traditional Medicine Store in Guangdong Province of China on 15 May 2018 and were identified by Chaojun Wang from Guangdong Medical University according to the Chinese Pharmacopoeia. Voucher specimen (GDMU-2018-0004) was deposited in the herbarium of the same institution. Foetal bovine serum (FBS), medium RPMI-1640, penicillin and streptomycin were purchased from Gibco (Grand Island, NY). The monosaccharide standards [glucose (Glc), galactose (Gal), rhamnose (Rha), arabinose (Ara), xylose (Xyl), mannose (Man), glucuronic acid (GlcA) and galacturonic acid (GalA)] at >98% purity, trifluoroacetic acid (TFA), 3-(4,5-dimethylthiazol-2-yl)-2,5-diphenyltetrazoliumbromide (MTT), T-series dextran (T-10, T-40, T-70, T-500, and T-2000) and Hoechst 33342 were obtained from Sigma Chemical Co. (St. Louis, MO). Sephadex G-100 and DEAE-cellulose were purchased from Amersham Pharmacia Co. (Uppsala, Sweden). The primary antibodies against cleaved caspase 3, Bax, Bcl-2, bone morphogenetic protein 2 (BMP2), Runt-related transcription factor 2 (RUNX2), Col I, OPG, ALP, β-actin and horseradish peroxidase (HRP)-conjugated secondary antibody were purchased from Santa Cruz Biotechnology (Santa Cruz, CA). Annexin V-FITC (fluorescein isothiocyanate)/PI (propidium iodide) kit was purchased from BD Biosciences (San Jose, CA). All other chemicals and reagents were of analytical grade and purchased from Sinopharm Chemical Reagent Company (Shanghai, China).

### Isolation and purification of polysaccharide SOWPa

The dried roots of *S. officinalis* were pulverized and passed through 100-mesh sieve. The pigments, impurities and lipophilic molecules in the dried powder were removed by the Soxhlet extraction method with 80% ethanol as solvent for two times (each for 2 h). Then, the residues were extracted with distilled water at 100 °C three times and 3 h each time. The whole aqueous extract was filtered, centrifuged (10,000 rpm, 20 min) and concentrated at 45 °C in a rotary evaporator followed by the addition of Sevag solution (chloroform:butyl alcohol, 4:1) to remove the dissociative protein. The resulting supernatant was concentrated and slowly mixed with anhydrous ethanol until the final alcohol concentration reached 80%, and then stand at 4 °C overnight to precipitate polysaccharides. The resulting precipitates were dissolved in a small amount of distilled water, followed by dialysis using a dialysis tube (MW cut-off 12,000) against tap water and distilled water for two days, respectively. The sample in the tube was combined, concentrated and precipitated with anhydrous ethanol as described above. After centrifugation (10,000 rpm, 20 min), the precipitation was successively washed with anhydrous ethanol and acetone in turn, and then dried to yield crude polysaccharides from the *S. officinalis* (SOP, 20.4 g).

The crude SOP (1 g) was dissolved in distilled water (5 mL) and the supernatant was fractionated on a DEAE-cellulose anion-exchange column (50 × 2.5 cm i.d.) eluting with distilled water and gradient NaCl solutions (0.2, 0.5 and 1 M) at a rate of 2 mL/min. All the fractions were collected by the automated fraction collector and combined as per carbohydrate contents in the collected tubes using the phenol-sulfuric acid method (Dubois et al. 1956), yielding three fractions: SOWP (distilled water), SOP2 (0.2 M NaCl) and SOP3 (0.5 M NaCl). The water eluate SOPW was further purified by size-exclusion chromatography (4 × 120 cm) on a Sephadex G-100 column with distilled water at a flow rate of 0.5 mL/min. The main resulting polysaccharides fraction SOWPa was collected, concentrated and dried for further use.

### Chemical compositions analysis

Content of carbohydrate was determined by phenol-sulfuric acid colorimetric method with glucose as a standard (Dubois et al. 1956). The protein content was quantified using the standard Bradford’s method (Bradford 1976), with bovine serum albumin as a standard. The uronic acid content was measured by *m*-hydroxybiphenyl method (Filisetti-Cozzi and Carpita 1991), with glucuronic acid as a standard.

### Molecular weight determination

The homogeneity and the average molecular weight of SOWPa was evaluated and determined using high performance gel permeation chromatography (HPGPC) on a Waters HPLC system, coupled with a TSK Gel G3000SWxl column (7.5 mm × 300 mm, 5 μm), a Waters 2410 differential refractive index detector (RID) and an on-line degaser. The polysaccharide sample dissolved in distilled water (10 mg/mL) was passed through a 0.22 μm filter and a 20 µL aliquot was injected for each run with 0.1 M MNa_2_SO_4_ mobile phase at a flow rate of 0.8 mL/min. The column was calibrated with T-series Dextran standards of known molecular weights (Dextran T-10, T-40, T-70, T-500 and T-2000) and the average molecular weight of polysaccharide fractions were estimated by reference to the equation of the calibration curve.

### Monosaccharide composition analysis

Gas chromatography (GC) was performed as described previously to determine the monosaccharide composition of the polysaccharide (Sun et al. 2008), with minor modifications. Briefly polysaccharide sample (10 mg) was hydrolysed with 2 M TFA (2 mL) for 6 h, at 100 °C. After the hydrolysis, the solution was evaporated with methyl alcohol (MeOH) under reduced pressure to remove TFA completely and the residue sample was conventionally acetylated with pyridine–acetic anhydride as described previously (Oades 1967; Johnes and Albersheim 1972). Finally, acetylated monosaccharide units were immediately analysed on an Agilent 7890A instrument fitted with a capillary column of HP-5 (30 m × 0.32 mm × 0.25 μm) and a flame ionization detector (FID). The column temperature was programmed at starting 120 °C for 3 min, and increased to 210 °C at a rate of 3 °C/min, and then maintained for 5 min. N_2_ was used as carrier gas and the flow rate was 1.0 mL/min. Monosaccharide standards (Glc, Gal, Rha, Ara, Xyl, Man, GlcA and GalA) and internal standard inositol were used to determine the monosaccharide composition and co-responding molar percentages in the same way. All measurements were repeated three times.

### PRP preparation

All experiments were approved by the Institutional Animal Care and Use Committee of the First Affiliated Hospital of Jinan University. Four male healthy New Zealand white rabbits with an average age of 6 months and an average weight of 4.3 kg were used for rabbit PRP production according to the method of Aghaloo (Freymiller and Aghaloo 2004), with minor modification. Briefly, 10 mL samples of peripheral blood were drawn from the marginal ear vein of rabbits under anaesthesia, and then placed into sterile conical tube containing 1.5 mL of sodium citrate before being centrifuged at 300×*g* for 10 min to separate the plasma from the red blood cells. The resulting plasma above the albuginea layer was collected in another centrifugal tube and centrifuged again at 800×*g* for 15 min at 4 °C. After removing platelet-poor plasma on the uppermost layer, the remaining 1.0 mL PRP was stored at −80 °C before use. The PRP volume extracted from each rabbit was 9.0 ± 0.35 mL.

### ACL fibroblasts preparation

ACL fibroblasts were harvested from rabbit ACL using sterile technique. In brief, ACL tissue were chopped into pieces and digested with 0.25% trypsin/EDTA at 37 °C for 1 h. Then, this mixture was centrifuged at 1000×*g* for 20 min and subsequently cultured in completed Dulbecco’s modified Eagle medium (DMEM) supplemented with 10% FBS at 37 °C in 5% CO_2_ humidified incubator in the absence of antibiotics. Then, the first passage cells were expanded and passaged until the third time, which were used for all experiments.

### Cell viability assay

The cell viability was evaluated using the colorimetric MTT assay, which is based on the conversion of yellow MTT to the purple formazan derivatives by mitochondrial enzymes in viable cells (Torres-González et al. 2011). Briefly, ACL fibroblasts were planted into 96-well flat-bottomed plates at a density of 1.0 × 10^4^/well and allowed to adhere to the plastic plates for 24 h before treatment. Afterwards, cells were treated with SOWPa (25 and 100 mg/kg), PRP, PRP + SOWPa (25 and 100 mg/kg) or vehicle alone for 24, 48 or 72 h. Then, 10 µL MTT solution (5 mg/mL) was added to each well in the dark at 37 °C for another 4 h before adding 150 μL of DMSO to fully dissolve the blue formazan crystals in living cells. Finally, the average value of optic density was measured using an ELISA reader (Bio-Rad model 550, Hercules, CA) at a wavelength of 450 nm. The cell survival rate (%) was defined as the ratio of absorbance of treated cells to untreated control cells. Each experiment was conducted in triplicate and repeated three times.

### Nuclear staining with Hoechst 33342

The morphological alteration of cells was evaluated by Hoechst fluorescence 33342 staining assay and performed as reported earlier (Liu et al. 2012). In short, ACL cells (1 × 10^6^ cells/mL) were seeded onto glass coverslips in six-well culture plate, and then treated with SOWPa (25 and 100 mg/kg), PRP, PRP + SOWPa (25 and 100 mg/kg) or vehicle alone for 72 h. The cells were subsequently washed with PBS, fixed in 4% paraformaldehyde for 30 min, and stained with Hoechst 33342 dye (5 μg/mL) in the dark for 10 min at 37 °C. After removal of excessive staining dye with three washes with PBS, morphological analysis was performed using a fluorescence inverted microscope (IX-71, Olympus, Tokyo, Japan).

### Flow cytometry

The extent of cell apoptosis was measured using Annexin V Apoptosis Detection kit, which was performed using flow cytometry to quantify the levels of detectable phosphatidylserine (PS) externalization on the outer membrane of apoptotic cells (Evens et al. 2004). Briefly, after the incubation, both adherent and floating ACL cells were collected, washed twice with PBS, and stained with 5 μL of annexin V-FITC and 5 μL of PI (50 μg/mL) in dark for 15 min following the instruction provided by the manufacture. Thereafter, at least 1 × 10^4^ cells in each sample were immediately collected and measured on a Becton-Dickinson FACS-Calibur flow cytometer using Cell Quest software to detect apoptotic cells (annexin V^+^/PI^−^) and necrotic cells (annexin V^+^/PI^+^). For each analysis, 30,000 events were recorded and the apoptosis rate (%) was calculated on the sum of (Annexin V^+^/PI^−^) and (Annexin V^+^/PI^+^) cells.

### Cell migration assay

Cell migration was conducted using a transwell chamber coated with a pore size of 8 μm polyethylene terephthalate filters according to the methods described by Lin et al. (2019). After different treatment, surviving cells were seeded into the top chamber at 2 × 10^4^ cells/well in serum free medium and the bottom chamber was filled with DMEM containing 10% FBS as chemoattractant. After incubation for 24 h at 37 °C, the migrated cells on the lower side of the filter were fixed, stained with crystal violet and counted from at least six random fields under a light microscope (Olympus, Tokyo, Japan). Each experiment was repeated three times.

### Western blot analysis

Western blot analysis was performed to determine the expression of different proteins involved in apoptosis. Following the various incubations, cells were harvested, washed with cold PBS (pH 7.4), and lysed with extraction buffer (25 mM Tris–HCl, pH 7.5, 1 mM EDTA, pH 8.0, 150 mM NaCl, 1% Triton X-100, 0.1% Nonidet P-40, 1 mM PMSF and 1 mM protease inhibitor cocktail) on ice for 30 min. The lysates were centrifuged at 10,000×*g* at 4 °C for 5 min and the protein concentrations in the supernatants were determined by the method of Bradford. For Western blot analysis, equal amounts of protein (25–50 μg) was subjected to 12% SDS polyacrylamide (PAGE) gel electrophoresis and electrophoretically transferred onto a nitrocellulose membrane. The membrane was blocked with 5% powdered non-fat milk for 1 h to block non-specific protein binding, and then incubated with the primary antibodies against Bax, Bcl-2, cleaved-caspase-3, Runt-related transcription factor 2 (RUNX2), alkaline phosphatase (ALP), BMP2, collagen I (Col I), osteoprotegerin (OPG) and β-actin overnight at 4 °C. After washing with TBS-Tween 20 (TBST) thrice, the blots were incubated with s HRP-conjugated secondary antibody for 1 h at room temperature in blocking solution, and washed thrice with TBST. Protein bands were visualized. The detection of protein bands was visualized by enhanced chemiluminescence (ECL) kit according to the recommended procedure.

### Statistical analysis

Data are presented as mean ± standard deviation (S.D.). Statistical analysis of data was performed with one-way analysis of variance (ANOVA) followed by Student’s *t*-test using the GraphPad Prism 5 System (GraphPad Software, La Jolla, CA). *p* Value less than or equal to 0.05 was considered statistically significant.

## Results

### Physicochemical properties of SOWPa

**Figure 1.**
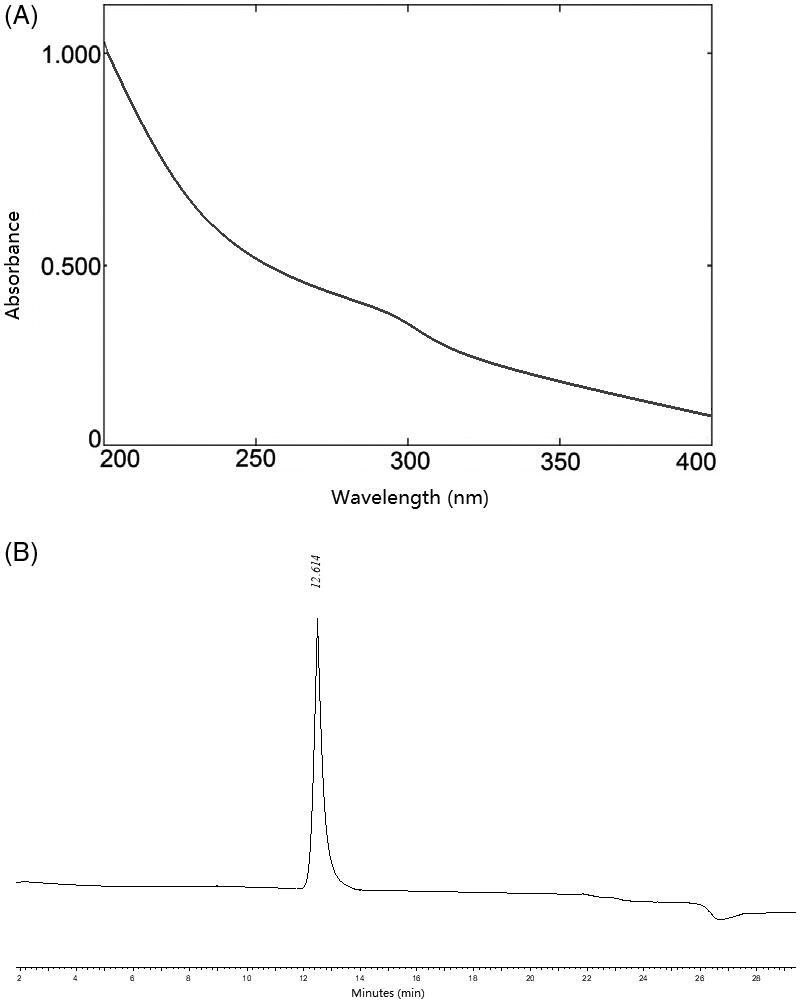
(A) The UV spectra of SOWPa. (B) The HPGPC profile of SOWPa.

A water-soluble polysaccharide SOWPa was purified from the roots of *S. officinalis*, by lipid removal with 80% ethanol, hot water extraction, ethanol precipitation, DEAE-cellulose ion exchange chromatography and Sephadex G-100 gel filtration chromatography with a yield of 1.47 g, accounting for 0.15% of the dried material (980 g). SOWPa contains 94.53% carbohydrate content and no protein or uronic acid was detected in it. Moreover, UV scanning of SOWPa in Figure 1(A) showed no absorbance at 260 and 280 nm, indicating that there was no protein and nucleic acid in SOWPa, which was consistent with the chemical analysis. The HPGPC profile of SOWPa was a single and symmetrically sharp peak, indicating that SOWPa was a homogeneous polysaccharide. To calculate the MW of polysaccharides, the calibration curve was created by different of standard Dextran from 9.6 to 714.5 kDa and plotted as the molecular weights on a log scale versus the retention time, to yield a standard regression curve equation as follows: log Mw= −0.1433*t* + 7.1245, *R*^2^=0.9969 (*t* is retention time). According to the retention time (12.614 min) in Figure 1(B), the average MW for SOWPa was estimated to be 2.07 × 10^5^ Da. GC analysis showed SOWPa consisted of glucose and galactose in a molar ratio of 2.01:1.01.

### Influence of PRP, SOWPa (100 mg/kg) or PRP + SOWPa (100 mg/kg) on cell viability of ACL fibroblasts

**Figure 2.**
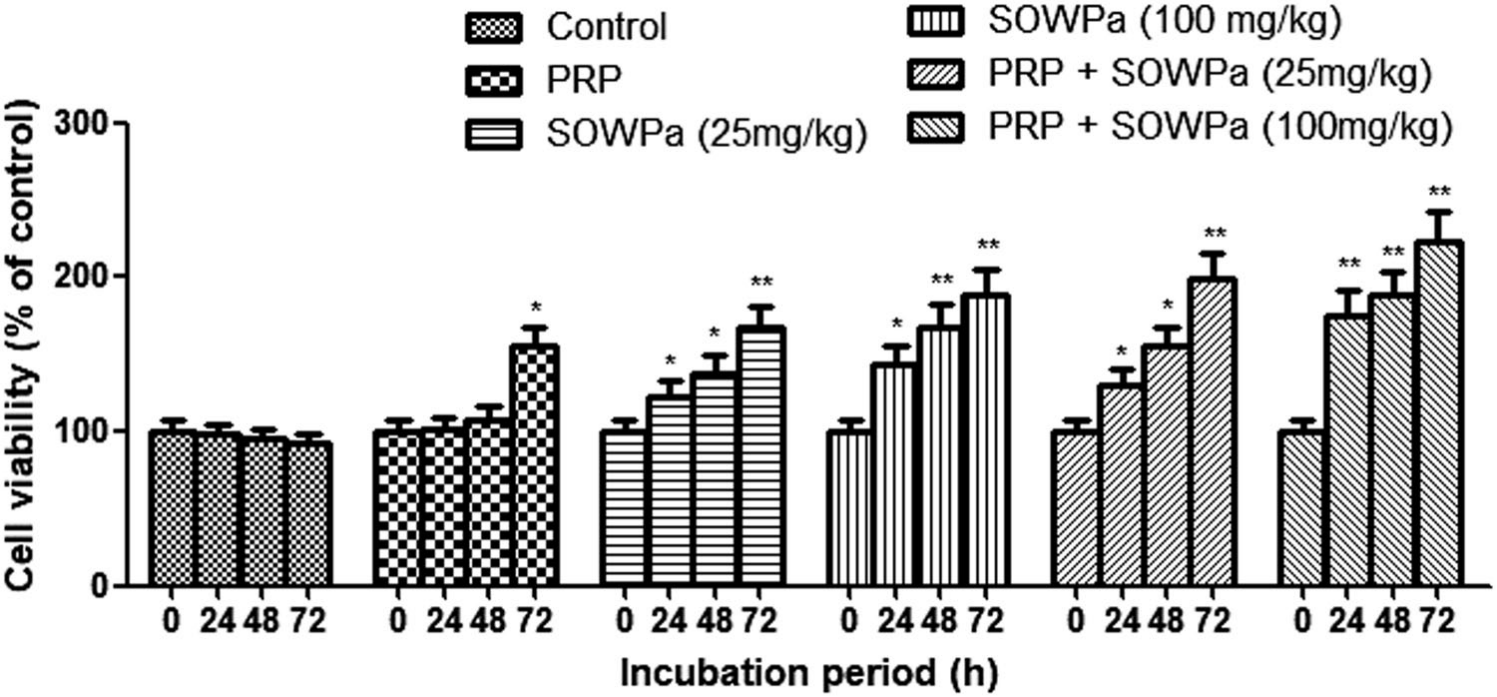
The effect of PRP, SOWPa (25 and 100 mg/kg) and their combination on the cell viability of ACL fibroblasts at 24, 48 and 72h. The data are expressed as the mean ± standard deviation (SD) of triplicate samples. *p < 0.05, **p < 0.01, compared with the control.

In a first set of experiments, we sought to characterize the influence of PRP, SOWPa or their combination on cell viability of ACL fibroblasts in different conditions. It was found that all cells displayed no significant changes in cell viability following 24 and 48 h co-culture with PRP or without (Figure 2). However, the cell viability was increased in the presence of SOWPa (25 and 100 mg/kg) at 24, 48, and 72 h. The same trend was also observed, but with higher cell viability rate in ACL fibroblasts treated with PRP + SOWPa (especially at 100 mg/kg) than either SOWPa or PRP treatment at any time. In view of these results, we chose 72 h and SOWPa at 100 mg/kg as optimal parameters in the following assays.

### Influence of PRP, SOWPa (100 mg/kg) or PRP + SOWPa (100 mg/kg) on migration of ACL fibroblasts

**Figure 3.**
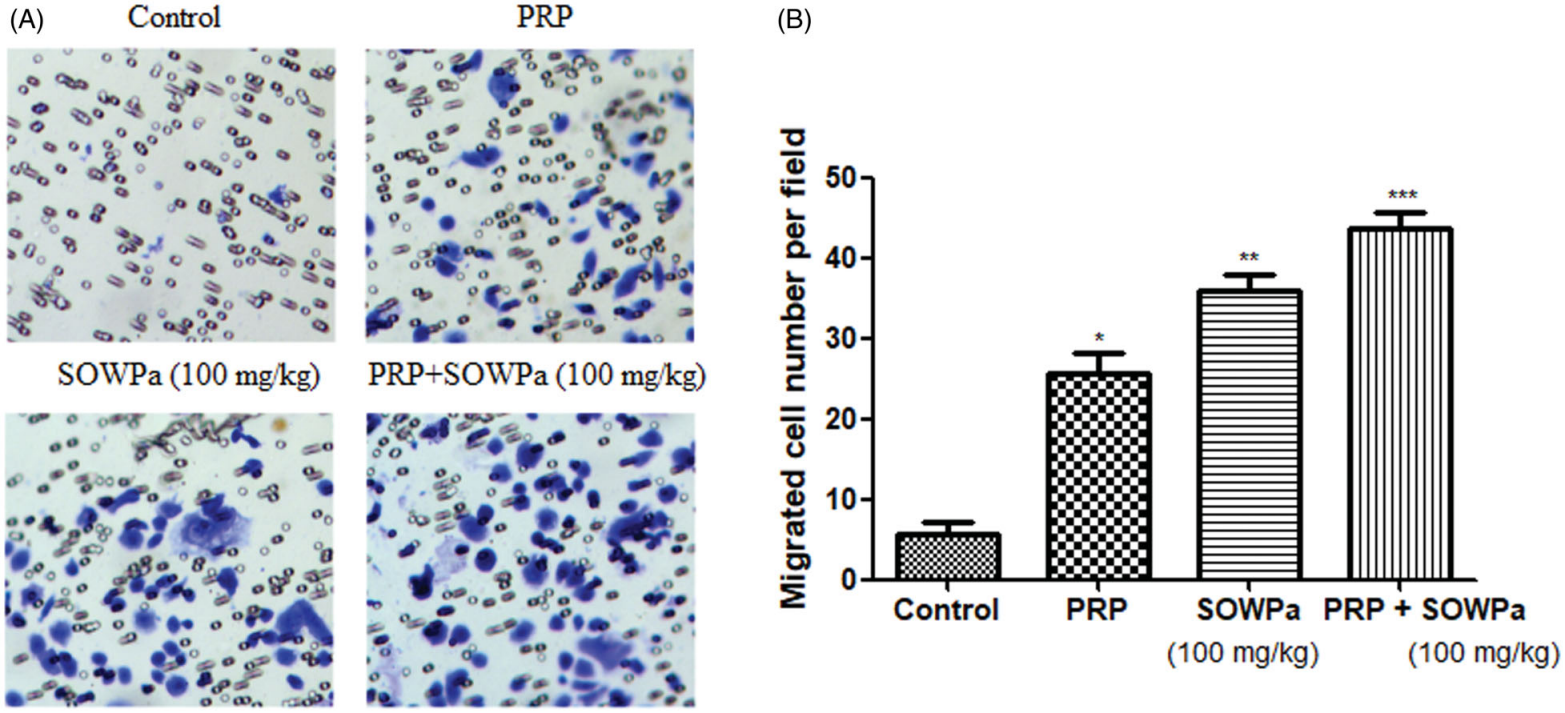
The effect of PRP, SOWPa (100 mg/kg) and their combination on the cell migration of ACL fibroblasts at 72h. The data are expressed as the mean ± standard deviation (SD) of triplicate samples. *p < 0.05, **p < 0.01, ***p < 0.001, compared with the contro.

To explore the effect of PRP, SOWPa (100 mg/kg) or PRP + SOWPa on the motility of ACL fibroblasts, cell migration ability was measured using transwell assay. As illustrated in Figure 3(A,B), it was found that PRP or SOWPa (100 mg/kg) significantly increased cell migration at 72 h as compared the control cells (*p* < 0.05), but the effects become much more pronounced in ACL fibroblasts in response to both PRP and SOWPa (100 mg/kg) treatment (*p* < 0.01).

### Influence of PRP, SOWPa (100 mg/kg) or PRP + SOWPa (100 mg/kg) on apoptosis of ACL fibroblasts

**Figure 4.**
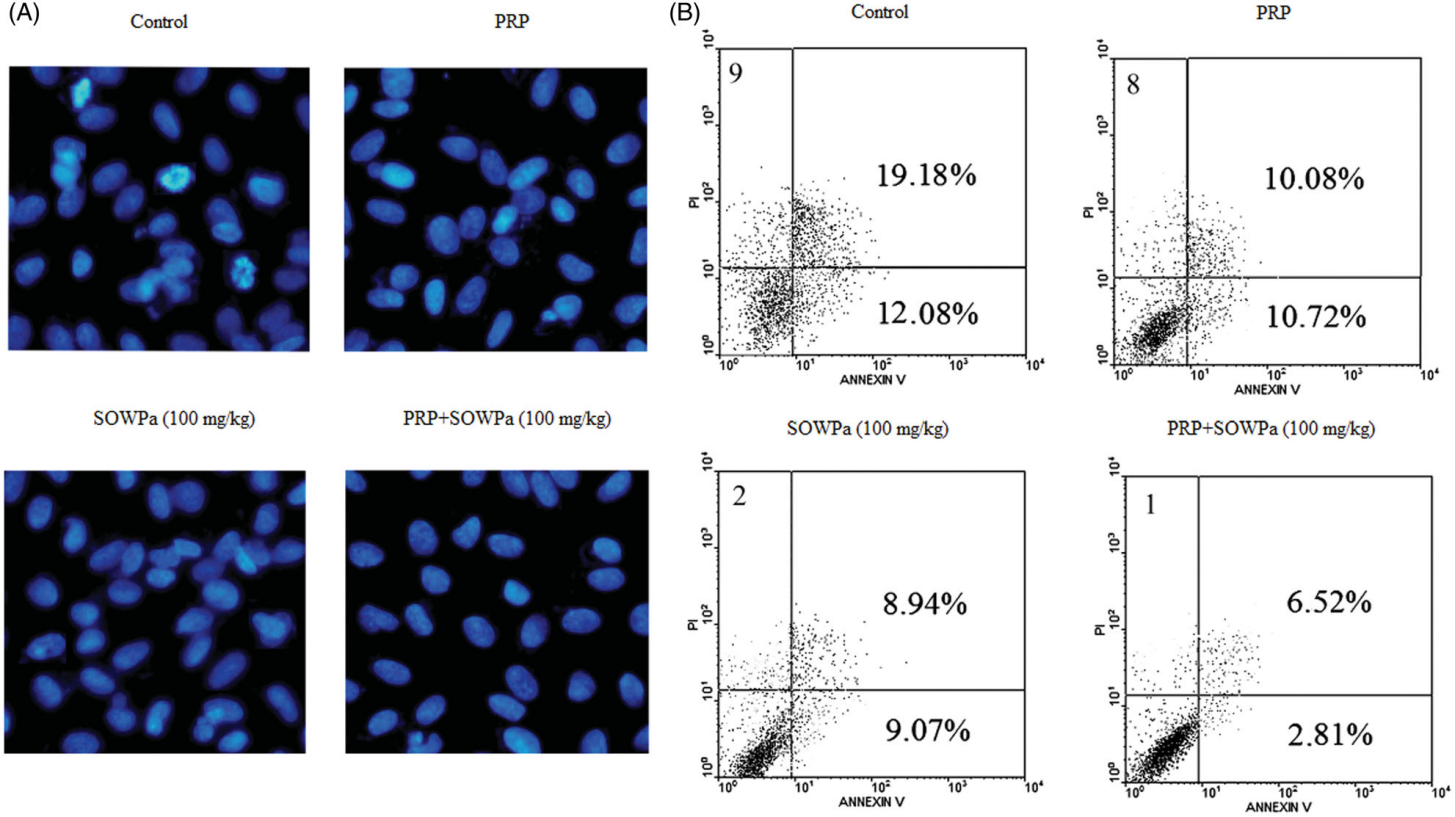
(A) Hoechst 33342 fluorescence staining of ACL fibroblasts after treatment with PRP, SOWPa (100 mg/kg) or PRP + SOWPa (100 mg/kg) for 72 h. (B) Flow cytometry analysis of ACL fibroblasts after treatment with PRP, SOWPa (100 mg/kg) or PRP + SOWPa (100 mg/kg) for 72 h.

To investigate whether this cell proliferation effect was related to cell apoptosis, the morphological alterations of ACL fibroblasts were measured. Staining of cells with Hoechst 33342 revealed chromatin condensation in control cells (Figure 4(A)). In contrast, no apoptotic nuclei were observed in ACL fibroblasts in response to PRP, SOWPa (100 mg/kg) or PRP + SOWPa (100 mg/kg) treatment for 72 h. To further confirm and quantify apoptosis ACL fibroblasts after treatment with PRP, SOWPa (100 mg/kg) or PRP + SOWPa (100 mg/kg), the cells were stained with Annexin V-FITC/PI and analysed by flow cytometry. The results suggested that three kinds of treatment dramatically decreased the cell apoptosis of ACL fibroblasts, with apoptosis rate of 31.26 ± 2.35% for control, 20.80 ± 1.89% for PRP, 18.01 ± 1.55% for SOWPa (100 mg/kg) and 9.33 ± 0.78% for PRP + SOWPa (100 mg/kg), respectively (Figure 4(B)).

### Influence of PRP, SOWPa (100 mg/kg) or PRP + SOWPa (100 mg/kg) on osteogenic differentiation ability of ACL fibroblasts

**Figure 5.**
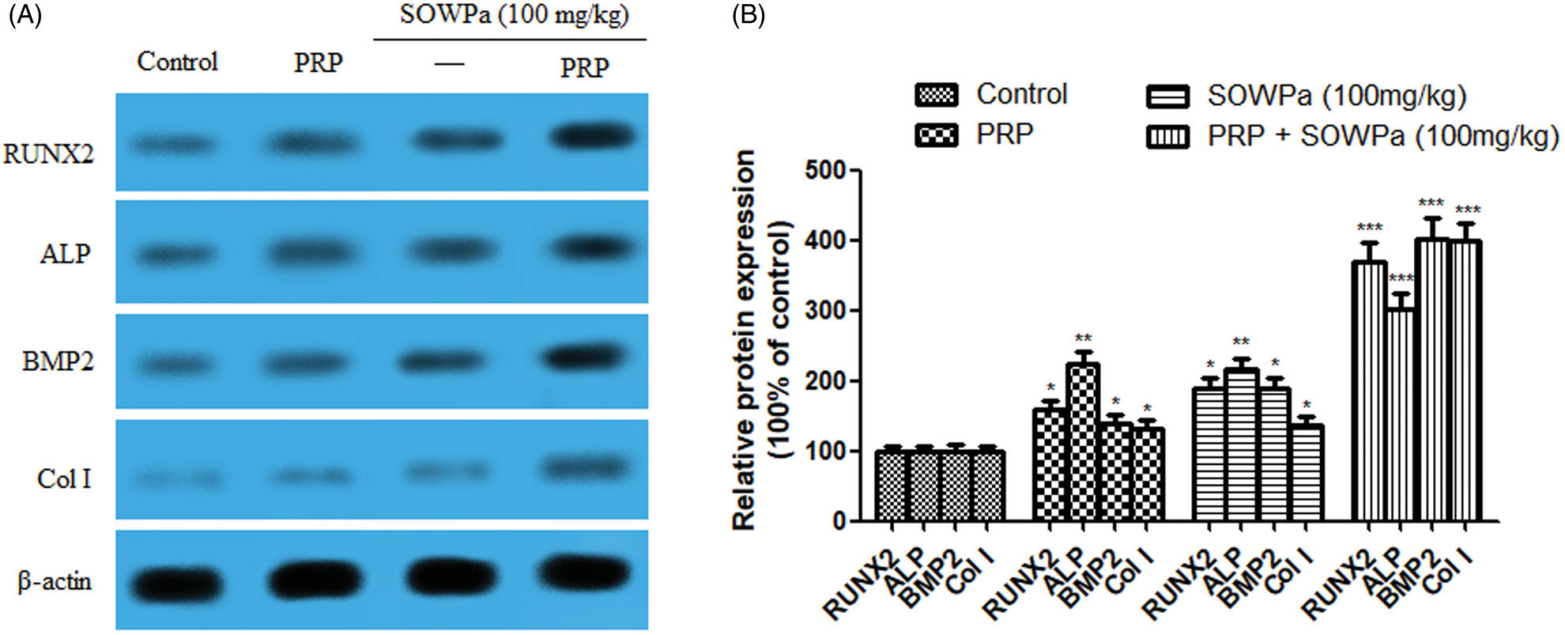
(A) The effect of PRP, SOWPa (100 mg/kg) and their combination on the protein expression of differentiation markers, such as RUNX2, ALP, BMP2, and Col I of ACL fibroblasts at 72h. (B) Quantitative analysis of the protein expression. The data are expressed as the mean ± standard deviation (SD) of triplicate samples. *p < 0.05, **p < 0.01, ***p < 0.001, compared with the control.

To further determine whether PRP, SOWPa (100 mg/kg) or PRP + SOWPa (100 mg/kg) could affect the osteogenic differentiation of ACL fibroblasts, the protein expression of differentiation markers, such as RUNX2, ALP, BMP2 and Col I were measured using Western blotting. It was first observed that RUNX2, ALP, BMP2, Col I and OPG protein expressions were slightly elevated in PRP or SOWPa (100 mg/kg)-treated cells and remarkably increased in PRP + SOWPa (100 mg/kg)-treated cells compared with control group (*p* < 0.05, 0.01 or 0.001, Figure 5(A,B)).

### Influence of PRP, SOWPa (100 mg/kg) or PRP + SOWPa (100 mg/kg) on TLR-4/NF-κB pathway of ACL fibroblasts

**Figure 6.**
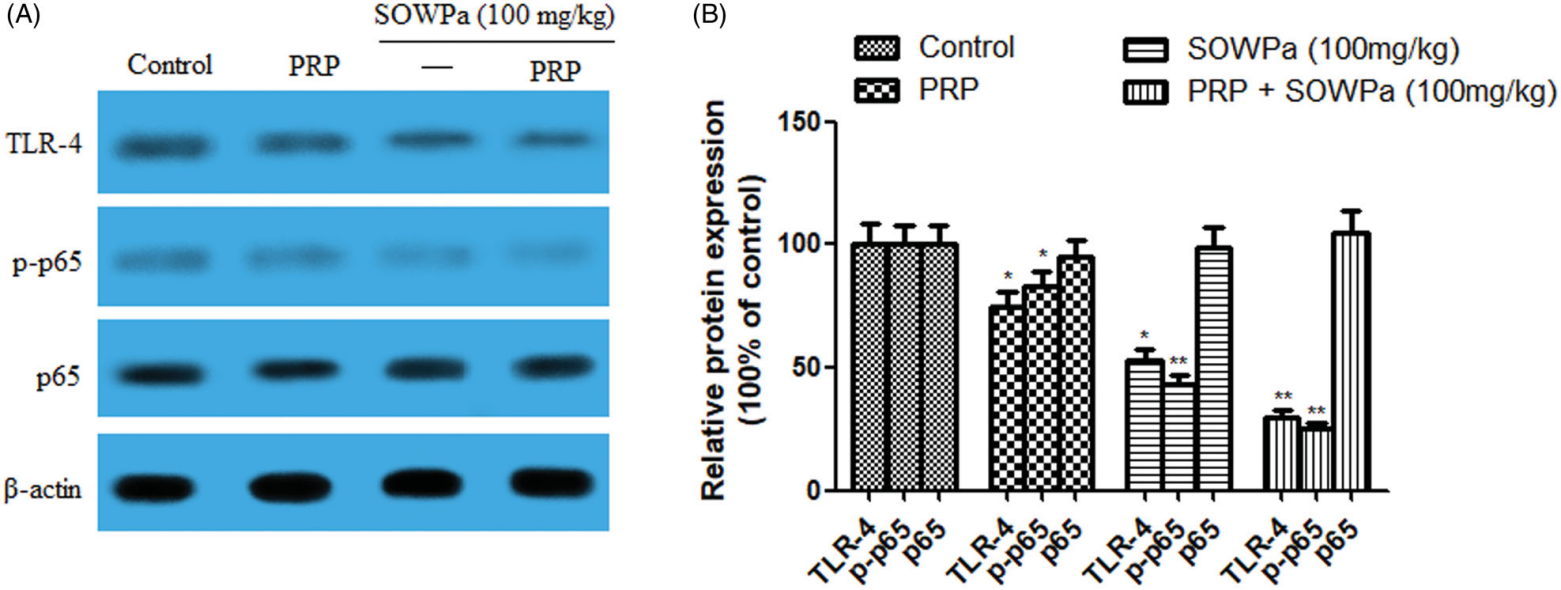
(A) The effect of PRP, SOWPa (100 mg/kg) and their combination on the protein expression of TLR-4, p-p65, and p65 of ACL fibroblasts at 72h. (B) Quantitative analysis of the protein expression. The data are expressed as the mean ± standard deviation (SD) of triplicate samples. *p < 0.05, **p < 0.01, compared with the control.

Blocking TLR-4/NF-κB pathway could improve osteogenic differentiation, cell viability and motility. Thus, TLR-4/NF-κB pathway was examined. As expected, TLR-4 and p65 phosphorylation were tremendously inhibited in ACL fibroblasts by PRP, SOWPa (100 mg/kg) or their combination, but p65 remained unchanged at all cases (Figure 6(A,B)). More importantly, the maximum inhibitory effect on TLR-4 and p65 phosphorylation occurred in PRP plus SOWPa treatment.

## Discussion

The ACL is an important structure to keep the knee stable (Fetto and Marshall 1980). The incidence of ACL injury remains high, especially among athletes (Renstrom et al. 2008). ACL fibroblasts are one of the most important cells in maintaining the integrity and homeostasis of the ACL and played a vital role during the wound healing process for ACL reconstruction surgery (Fan et al. 2009), which is a common intervention for this disorder. Due to its stem cell-like property, ACL fibroblasts have potent proliferation, differentiation and migration abilities (Vavken et al. 2010). Thus, improving the proliferation, differentiation and migration capacity may be one of the beneficial strategies to facilitate the fibrotic tissue regeneration after ACL reconstruction surgery. PRP have all shown the capacity to accelerate the healing of hard and soft tissues in ligament engineering constructs (Goh et al. 2003) and bone reconstruction (Dimitriou et al. 2005). Nevertheless, existing preclinical and clinical evidence regarding PRP use in ACL surgery failed to demonstrate a clear benefit. To overcome these problems, therefore, it is the first time that we evaluate if the addition of one polysaccharide SOWPa from the roots of *S. officinalis* would serve as the impetus to PRP for regenerative ability of ACL fibroblasts.

In the present study, we successfully isolated and purified a homogeneous polysaccharide from the roots of *S. officinalis* with a molecular weight of about 2.07 × 10^5^ Da. By comparing with the retention time and peak areas of the standards under the same conditions, GC analysis showed SOWPa was composed of glucose and galactose in a molar ratio of 2.01:1.01. Both proliferation and migration of ACL fibroblasts are essential for repair and regeneration (Woo et al. 2007). The results from current studies have demonstrated that PRP, SOWPa (100 mg/kg) or PRP + SOWPa could significantly promote the proliferation and migration of ACL fibroblasts, especially at 72 h post seeding. To investigate whether this cell proliferation effect was related to cell apoptosis, the morphological alterations and apoptotic rate of ACL fibroblasts were measured by Hoechst 33342 staining and flow cytometry, respectively. In line with these results, the percentage of apoptotic cells decreased greatly in ACL fibroblasts following treatment with PRP, SOWPa (100 mg/kg) or PRP + SOWPa. The peak value for cell viability, apoptosis and migration was all achieved in cells in response to both PRP and SOWPa (100 mg/kg) treatment. The above data indicated that the protective effect of PRP, SOWPa or both on the cell viability of ACL fibroblasts were mostly due to the inhibition of apoptosis and the combination exposure to PRP and SOWPa reached out to the maximum potency than either.

BMP2, Col I, OPG and ALP played a fundamental role in the regulation of cell differentiation (Boumah et al. 2005; Di Benedetto et al. 2017). Previous published studies have suggested that PRP showed a strong potential to favour osteogenic differentiation for soft-tissue regeneration (Griffin et al. 2009; Kobayashi et al. 2017). A similar result was drawn in the present study, the protein expression of these four differentiation markers was markedly elevated in ACL fibroblasts when treated with PRP (*p* < 0.01 or *p* < 0.05) or SOWPa (*p* < 0.01 or *p* < 0.05), especially with their combination (*p* < 0.001) as compared to the control cells. It is possible that PRP serves as the material for regeneration, while SOWPa serves as the impetus to promote appropriate differentiation, indicating that osteogenic differentiation ability of RPP on ACL fibroblasts could be enhanced by SOWPa treatment.

Recent studies have suggested that the inactivation of TLR-4 and NF-κB was involved in ECM synthesis and osteogenic differentiation (Wang et al. 2017; Zhang, Hu, et al. 2018). The results from the Western blot assay demonstrated that the expression of TLR-4 and P-65 phosphorylation was dramatically decreased in ACL fibroblasts by exposure to either PRP or SOWPa (100 mg/kg). Their inhibition reached the lowest expression in PRP + SOWPa combined treatment. However, no change of p65 protein expression was observed in all groups.

## Conclusions

The results from the present study demonstrated that SOWPa is able to assist PRP to decrease the apoptosis and increase the cell viability, migration and differentiation of ACL fibroblasts via blocking TLR-4/NF-κB pathway. Our study is valuable for unravelling the underlying mechanism of SOWPa as an adjuvant to enhance regenerative potential of PRP for ACL reconstruction surgery. In the future, the exact mechanisms will be further verified in animals.
